# Biliary Atresia Associated with Polysplenia Syndrome, Dextrocardia, Situs Inversus Totalis and Malrotation of Intestines

**Published:** 2014-01-01

**Authors:** Praveen Mathur, Rahul Gupta, Varsha Soni, Reyaz Ahmed, Ram Babu Goyal

**Affiliations:** Department of Paediatric Surgery, SMS Medical College, Jaipur.

**Keywords:** Polysplenia, Biliary atresia, Malrotation

## Abstract

Biliary atresia (BA) is a rare disease and the end result of a destructive, inflammatory cholangiopathy, leading to fibrosis and biliary cirrhosis. It is classified into syndromic variety with various congenital anomalies and non-syndromic (isolated anomaly). We present here a 1-month-old female child with the syndromic variety of BA associated with polysplenia syndrome, dextrocardia, situs inversus totalis and malrotation of intestines. She developed jaundice in the first week of life. Kasai operation was performed but she developed cholangitis and septicemia 2.5 months after surgery and succumbed later.

## INTRODUCTION

Biliary atresia is a destructive, idiopathic, and inflammatory cholangiopathy that affects intra and extra hepatic bile ducts leading to fibrosis and obliteration of the biliary tract and development of liver cirrhosis [1]. Biliary atresia has an incidence of 1 in 8000-16700 live births with a slight female predominance [2,3].

Two different forms of BA have been identified. Syndromic (fetal or embryonic) form with various congenital anomalies such as polysplenia, asplenia, cardiac defects, situs inversus, pre-duodenal portal vein, absence of retro-hepatic inferior vena cava, intestinal malrotation, annular pancreas, Kartagener’s syndrome, duodenal atresia, esophageal atresia, polycystic kidney, cleft palate and jejunal atresia. Non-syndromic BA has an isolated anomaly [3-6]. In 11 to 25% of cases of biliary atresia associated malformations are present and polysplenia constitute 10% of these associated anomalies [5]. Association of BA with polysplenia syndrome, dextrocardia, situs inversus totalis and malrotation of intestine is present in isolation or in various combinations [2-5,7]; however its association with all of them in the same patient is very rare and we present here an infant with such a rare association.

## CASE REPORT

A 1-month-old female child presented with jaundice, clay colored stools and dark yellow urine since the first week of life. There was no history of fever and cyanotic spells. She was born full term via a normal vaginal delivery and was first in birth order. There was no consanguinity among the parents. Physical examination revealed jaundice, normal facies and no organomegaly. She was hemodynamically stable. She was put on phenobarbitone and vitamin K on the lines of treatment for cholestatic jaundice. There was some improvement in color of stools and icterus after 5 days of therapy. In paraclinic evaluation, hemoglobin was 9.8 gm%, TLC 13,200/mm, total and direct bilirubin were elevated (total bilirubin 4.64 mg/dl, and direct bilirubin 2.5 mg/dl) and was in favor of cholestasis. Liver enzymes were elevated (SGOT-165 IU/L and SGPT-77 IU/L). Alkaline phosphatase was markedly raised, 1991 IU/L (normal up to 300). Total serum protein (5.2 gm%)and albumin(3.3 gm%) were below the normal range. Prothrombin time (PT) and INR were corrected. Radiographs of chest and abdomen revealed dextrocardia and liver shadow in the left side. Abdominal ultrasound showed liver in the left, stomach in the right side of abdomen and situs inversus. Spleen was not visible, instead multiple rounded masses along splenic vessels were presented on right side, suggestive of polysplenia. Gallbladder was not visualized on ultrasound of the abdomen and IHBR were not dilated. Triangular cord sign in liver hilum could not be elicited. CECT confirmed the above findings (Fig. 1) and revealed situs inversus totalis. With well tampered Hepatobiliary iminodiacetic acid (HIDA) scan no excretion of the radiopharmaceutical tracer was noted. This data was highly suggestive of biliary atresia (BA). Hepatoportoenterostomy (Kasai) operation was planned. At operation, the liver was seen on left side, and looked cirrhotic, gallbladder was absent, common hepatic duct, right and left hepatic ducts were atretic. Stomach was on right side. Multiple spleens / polysplenia (21 splenicules), floating and non-floating were present on the right side of the abdominal cavity (Fig. 2) and there was Typical variety of malrotation. The diagnosis of Biliary Atresia associated with polysplenia syndrome, dextrocardia, situs inversus totalis and malrotation of intestine was made.

**Figure:**
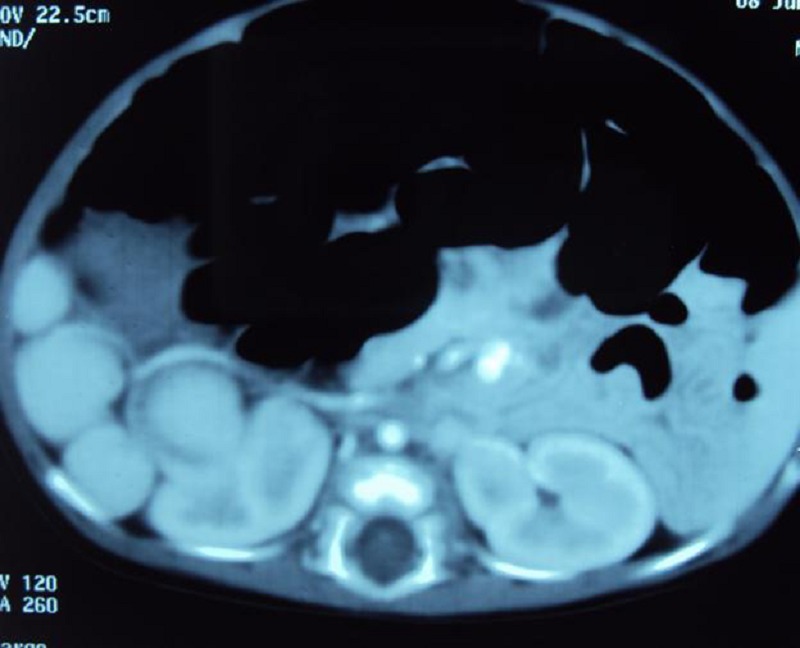
Figure 1: CT scan showing multiple splenicules.

**Figure:**
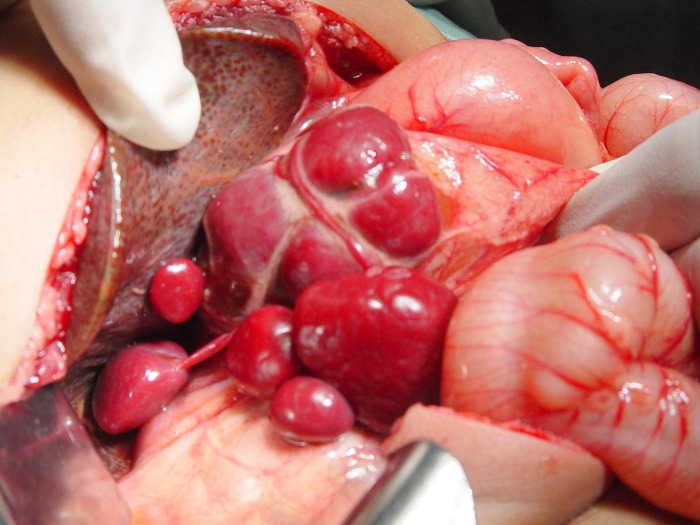
Figure 2: Intraoperative photograph showing polysplenia.

The atretic gallbladder and portal plate were dissected meticulously. Roux-en Y hepatico-jejunostomy was performed through the transverse colonic window. Malrotation was corrected by performing Ladd’s procedure. Liver biobsy was taken. Spleens were left as such. The postoperative recovery was uneventful. Patient was allowed oral feeding on 7th post-operative day and drain removed, the next day and discharged on 11th post-operative day. Stools gradually became cholic. Patient was started on ursodeoxycholic acid, prednisolone, fat soluble multivitamins, oral antibiotics and oral calcium supplementation. Repeat liver functions improved and patient was on regular follow up for one and half months. Thereafter child was lost to follow up. She reportedly had an episode of severe cholangitis and succumbed at a remote peripheral hospital after 2.5 months of surgery.

## DISCUSSION

Chandra in 1974 described a subclass of patients of polysplenia syndrome who had biliary atresia [3], but it was Davenport in the year 1993 who proposed the term Biliary Atresia Splenic Malformation (BASM) syndrome [4].

BA when associated with the polysplenia syndrome, is most likely caused by an early embryological insult during the phase of organ development, at approximately fifth week of intrauterine life [8]. The polysplenia syndrome is most common extrahepatic anomaly found in association with BA. Polysplenia syndrome is defined as polysplenia with variable association of number of anomalies i.e. heterotaxy of abdominal or thoracic organs, malrotation of gut, absent inferior vena cava, aberrant hepatic artery and preduodenal portal vein [4]. Syndromic infants were more likely to be female as is the non-syndromic group [4].

Situs inversus (SI) is a congenital anomaly characterized by the mirror-image orientation of the inner organs. The estimated incidence of SI is 1/15 000 to 1/10 000 live birth [9]. It results from failure of the developing embryo to establish normal left-right asymmetry. This results in malposition of thoraco-abdominal organs and vasculature, complex congenital heart and extracardiac abnormalities [10].

Abdominal heterotaxy and malrotation in a case of biliary atresia pose difficulties in the orientation of the roux-en-y loop [11]. In our case the stomach and spleens were lying on right side of the abdomen and there was malrotation, therefore roux loop was passed through a rent in the mesentery of colon with its orientation being a mirror image of the usual position for hepatico-portoenterostomy.

In a retrospective study, there were no differences in liver histology (e.g., degree of liver fibrosis) or in the HLA genotype between BASM and non-syndromic infants [5]. But BASM group has worse prognosis than non syndromic BA and whether this is caused by the presence of the other anomalies e.g., cardiovascular anomalies is debatable [4]. 

Biliary atresia polysplenia syndrome (BASM) is a very rare disease. Early diagnosis and appropriate treatment is very important to prevent development of liver cirrhosis. The operating surgeon must be aware of the surgical implications that may occur in case of associated malrotation and mirrored alimentary tract anatomy. It has a worse outcome than non-syndromic type. Compliance to the postoperative treatment is crucial to the outcome.

## Footnotes

**Source of Support:** Nil

**Conflict of Interest:** None

